# Supplementary material for: The interactome of intact mitochondria by cross-linking mass spectrometry provides evidence for coexisting respiratory supercomplexes
Source: Mol Cell Proteomics. 2017 Dec 8;17(2):216–32. doi: 10.1074/mcp.RA117.000470 (PMC5795388; doi:10.1074/mcp.RA117.000470)
Supplement: Supplemental Data [file supp_RA117.000470_133922_0_supp_23978_fzffwf.zip › spectra_annotation/mito_spectra_annotation/289-1-9-1-15-1.pdf]

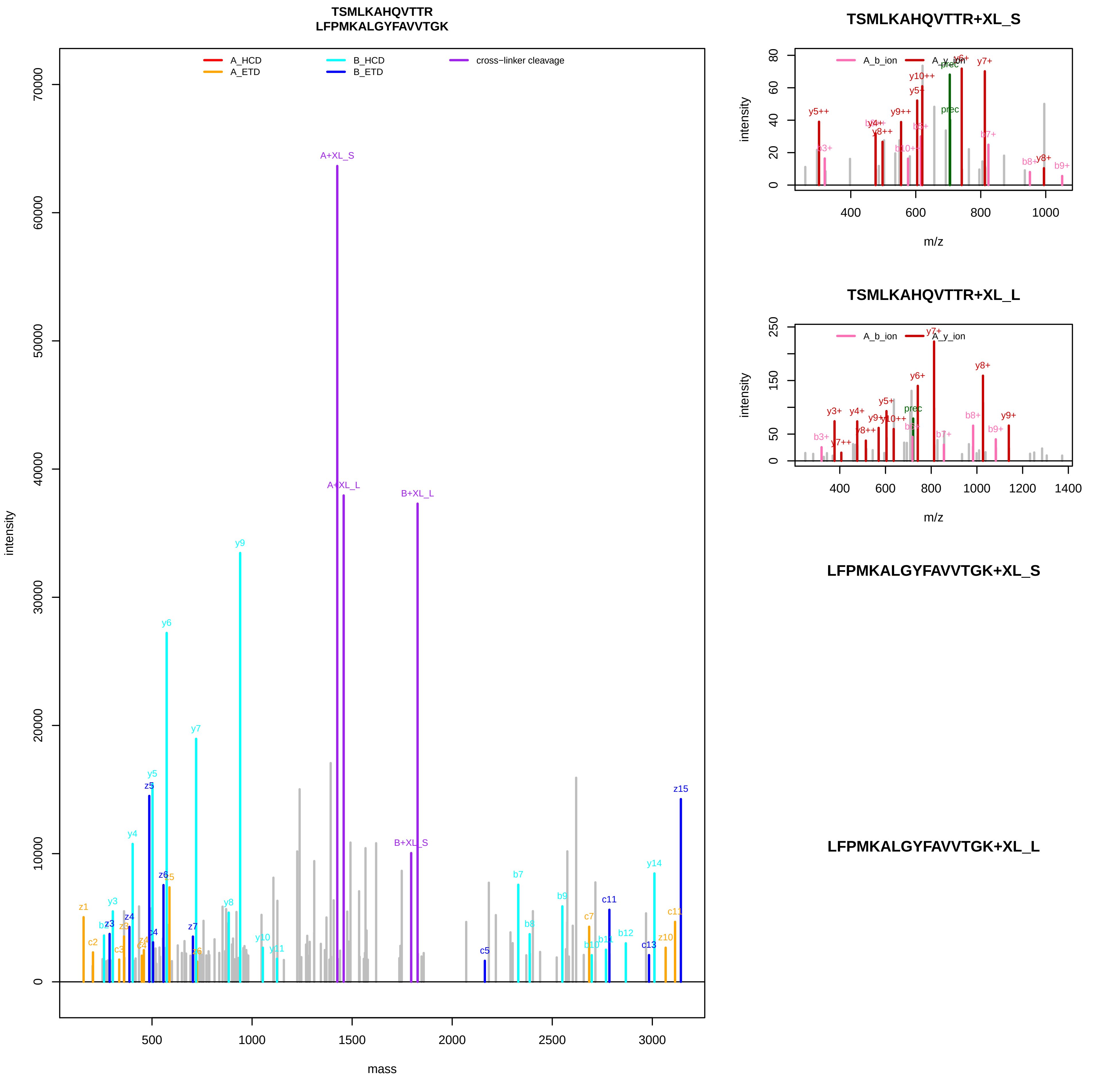
TSMLKAHQVTTR
LFPMKALGYFAVVTGK
A_HCD
A_ETD
B_HCD
B_ETD
cross−linker cleavage
intensity
mass
0
10000
20000
30000
40000
50000
60000
70000
500
1000
1500
2000
2500
3000
A+XL_S
A+XL_L
B+XL_L
B+XL_S
y9
y6
y7
y5
z5
y4
y3
y8
y10
y11
z1
c2
z3
z4
c4
z6
c5
z7
c5
b7
b8
b9
c7
b10
b11
c11
b12
c13
c10
c11
z10
y14
z15
TSMLKAHQVTTR+XL_S
A_b_ion
A_y_ion
intensity
m/z
prec
y5++
y10++
y5+
y9++
y8++
y7+
y6+
b3+
b7+
b8+
y8+
b9+
TSMLKAHQVTTR+XL_L
A_b_ion
A_y_ion
intensity
m/z
y7+
y8+
y6+
y5+
y3+
y4+
y9+
y10++
y8++
y7++
b3+
b8+
b9+
b7+
prec
LFPMKALGYFAVVTGK+XL_S
LFPMKALGYFAVVTGK+XL_L
